# Associations of gut-flora-dependent metabolite trimethylamine-N-oxide, betaine and choline with non-alcoholic fatty liver disease in adults

**DOI:** 10.1038/srep19076

**Published:** 2016-01-08

**Authors:** Yu-ming Chen, Yan Liu, Rui-fen Zhou, Xiao-ling Chen, Cheng Wang, Xu-ying Tan, Li-jun Wang, Rui-dan Zheng, Hong-wei Zhang, Wen-hua Ling, Hui-lian Zhu

**Affiliations:** 1 School of Public Health, Sun Yat-sen University, Guangzhou (510080), People’s Republic of China; 2 Faculty of Public Health, School of Medicine, Jinan University, Guangzhou (510632), People’s Republic of China; 3 Research and Therapy Center for Liver Disease, the Affiliated Dongnan Hospital of Xiamen University, Zhangzhou (363000), People’s Republic of China; 4 Department of Hepatobiliary Surgery, Sun Yat-Sen Memorial Hospital, University of Sun Yat-Sen, Guangzhou (510120), People’s Republic of China

## Abstract

Many studies suggest that trimethylamine-N-oxide (TMAO), a gut-flora-dependent metabolite of choline, contributes to the risk of cardiovascular diseases, but little is known for non-alcoholic fatty liver disease (NAFLD). We examined the association of circulating TMAO, choline and betaine with the presence and severity of NAFLD in Chinese adults. We performed a hospital-based case-control study (CCS) and a cross-sectional study (CSS). In the CCS, we recruited 60 biopsy-proven NAFLD cases and 35 controls (18–60 years) and determined serum concentrations of TMAO, choline and betaine by HPLC-MS/MS. For the CSS, 1,628 community-based adults (40-75 years) completed the blood tests and ultrasonographic NAFLD evaluation. In the CCS, analyses of covariance showed adverse associations of ln-transformed serum levels of TMAO, choline and betaine/choline ratio with the scores of steatosis and total NAFLD activity (NAS) (all *P*-trend <0.05). The CSS revealed that a greater severity of NAFLD was independently correlated with higher TMAO but lower betaine and betaine/choline ratio (all *P*-trend <0.05). No significant choline-NAFLD association was observed. Our findings showed adverse associations between the circulating TMAO level and the presence and severity of NAFLD in hospital- and community-based Chinese adults, and a favorable betaine-NAFLD relationship in the community-based participants.

Non-alcoholic fatty liver disease (NAFLD) is the most common liver disease in Western and Asian countries^1^. It may progress to chronic liver disease, culminating in end-stage liver disease. The prevalence of NAFLD in China is increasing at an alarming rate and currently ranges from 11.5%–27%^2^,^3^. Although the exact causes of this disease are uncertain, many studies have shown that food components and their metabolites may play important roles in its development and progression^4^.

Choline is an essential nutrient that serves as a component of phosphatidylcholine (PC), a precursor of the neurotransmitter acetylcholine. Choline can be oxidized to betaine in humans. Choline and betaine function as methyl donors in pathways involving the re-methylation of homocysteine to methionine to diminish blood homocysteine^5^ and in DNA and histone methylation, which may play potential roles in NAFLD and other cardio-metabolic diseases^6^. A few studies have examined the association of choline and betaine with fatty liver disease in animals and humans. Raubenheimer *et al.* have found that a choline-deficient diet greatly exacerbates fatty liver induced by high-fat diet consumption in rodents^7^. A small number of human studies have shown that the consumption of a low-choline diet promotes fatty liver and liver damage^8^,^9^. However, some epidemiological studies have found that a high blood choline level is positively associated with NAFLD^10^, metabolic syndrome (or dyslipidemia)^11^ and major adverse cardiovascular events (MACEs)^12^,^13^. It is unclear whether choline itself or its metabolite, which is produced by humans and gut microbiota, contributes to the adverse events experienced by individuals with high choline intake.

Choline can be metabolized to trimethylamine (TMA) by the gut microbiota (Fig. 1). TMA is subsequently oxidized by hepatic flavin-containing monooxygenases in the liver, forming trimethylamine-N-oxide (TMAO), which is then released into circulation^14^,^15^. Previous studies have revealed that TMAO may affect lipid absorption and cholesterol homeostasis and modulate glucose and lipid metabolism by decreasing the total bile acid pool size^16^. An animal study has suggested that TMAO may exacerbate impaired glucose tolerance by blocking the hepatic insulin signaling pathway and promoting the development of fatty liver in mice fed a high-fat diet^17^. Several human studies have shown that an elevated level of TMAO may increase the risk of cardiovascular disease (CVD) and exacerbate the effects of high choline and betaine levels on the risk of MACEs in a large-scale clinical cohort^14^,^18^. These studies have suggested that high circulating TMAO, a gut-flora-dependent choline metabolite, may increase the risk of cardiometabolic disease. However, to our knowledge, no study has examined the association between TMAO and NAFLD in humans to date, and few studies have determined the relationship between blood betaine and NAFLD in humans either.

The aims of the present study were to evaluate the association of circulating TMAO, choline and betaine with the presence and severity of NAFLD in clinical and community-based Chinese adults.

## Participants and Methods

### Study participants

We conducted a case-control study (CCS) and cross-sectional study (CSS). For the CCS, we recruited 60 adults (age: 18–60 years) with biopsy-proven NAFLD from outpatients with NAFLD evaluated firstly by ultrasonography, in addition to 35 controls with the same age rage at the Affiliated Dongnan Hospital of Xiamen University and Sun Yat-Sen Memorial Hospital from April 2012 to June 2013. The controls were patients who had undergone partial hepatectomy due to hepatic hemangioma or hepatolithiasis and had normal liver histopathology. Subjects with the following conditions were be excluded: confirmed heart diseases, stroke, cancer, excessive alcohol consumption, autoimmune liver disease or other conditions^19^ that may result in fatty liver. For the CSS, participants were drawn from a community-based cohort, from which 3,169 participants (40–75 years) were recruited among apparently healthy residents of Guangzhou, China between July 2008 and June 2010^20^. Of them, 1,996 participants without the following exclusion criteria underwent testing to determine the plasma TMAO, choline and betaine levels: a history of excessive alcohol consumption, the use of steatogenic medication, autoimmune liver disease, chronic viral hepatitis, and hepatic carcinoma or decompensation. In addition, 1,628 of the 1996 subjects underwent further NAFLD testing by ultrasonography between April 2011 and January 2013. Baseline data, including general information, plasma levels of TMAO, choline and betaine, and NAFLD measurements at follow-up were used in the CSS. The abovementioned exclusion criteria were used in both studies. The study protocol complied with all provisions of the 1975 Declaration of Helsinki and its current amendment, and was approved by the Ethics Committee of the School of Public Health of Sun Yat-sen University. Written informed consent was obtained from all participants.

### Data collection

For both the CCS and CSS, we collected general information using the same structured questionnaires and performed body measurements and biochemical tests of fasting serum lipid and glucose levels and plasma levels of TMAO, choline and betaine. Histopathological evaluation of liver biopsy samples and blood AST and ALT tests were conducted for the CCS, and ultrasonographic evaluation of NAFLD was performed in the CSS.

### Questionnaire interview and Anthropometric measurements

Face-to-face interviews were conducted to collect the following information: socio-demographic characteristics (e.g., age, gender, level of education, occupation and other factors), lifestyle habits (e.g., consumption of alcohol, tobacco and tea), physical activities, and history of chronic diseases and medication^20^. Body weight and height and waist circumferences (WC) were measured while the participants were barefoot and wearing light clothing. Body mass index (BMI, kg/m^2^) was also calculated. Two consecutive blood pressure (BP) measurements were performed on the right arm after the participant had been sitting for at least 10 min, and the mean value was used in the further analyses.

### Laboratory analysis

Fasting serum was isolated and stored at −80 °C until analysis. Serum concentrations of TMAO, choline and betaine were quantified by high-performance liquid chromatography with online electrospray ionization tandem mass spectrometry (HPLC-MS/MS) (Agilent 6400 Series Triple Quad LCMS; CA, USA)^20^. A volume of 60 μl of either the serum sample or standards was combined with 100 μl of acetonitrile containing 10 μM of internal standards [d9-choline, d9-betaine (Sigma-Aldrich, St. Louis, USA) and 9-TMAO (Toronto Research Chemicals Inc, Toronto, Canada)], and the sample was centrifuged at 13,000 × g for 10 min to precipitate the proteins. Finally, after an additional centrifugation step, the supernatant was analyzed after injection into a normal-phase silica column (2.1 mm × 100 mm, 5 μm) and equilibrated with 30% solution A (15 mmol/L ammonium formate in water, pH 3.0) and 70% solution B (acetonitrile) under isocratic elution with the flow rate of 0.2 mL/min. The coefficients of variation for the between-run assays were 6.0%, 4.91% and 6.21% for TMAO, choline and betaine, respectively.

Overnight fasting serum total cholesterol (TC), triglyceride (TG), LDL cholesterol (LDLc), HDL cholesterol (HDLc) and fasting blood glucose were measured by colorimetric methods using a Hitachi 7600-010 automated analyzer.

### Histopathologic evaluation

Liver biopsy samples were stained using both hematoxylin and eosin (H&E) and Masson’s trichrome methods. Histological assessment was performed according to the nonalcoholic steatohepatitis (NASH) Clinical Research Network Scoring System^21^ by two experienced pathologists who were unaware of the participants’ information. Fatty liver was categorized as follows, according to the proportion of steatosis^22^: grade 0 (<5%), 1 (5%–33%), 2 (33%–66%), or 3 (>66%). Hepatocellular ballooning was graded as none (grade 0), few balloons (grade 1), and many cells/prominent (grade 2). According to the number of inflammatory foci per field of view at a magnification of 200×, lobular inflammation was classified as grade 0 (none), 1 (<2 foci/field), 2 (2–4 foci/field) and 3 (>4 foci/field). The total NAFLD activity score (NAS) was calculated as the sum of the scores for steatosis, hepatocellular ballooning and lobular inflammation and was then categorized as no NASH (<3), borderline NASH (3−4) and NASH (≥5).

### Abdominal ultrasonography and Diagnosis of NAFLD

Ultrasonography of the upper abdominal organs was performed using a color Doppler ultrasound (Sonoscape SSI-5500, Shenzhen, China) with a 3.5 MHz probe by experienced physicians who blinded to the participants’ information. Fatty liver disease was diagnosed according to Graif’s criteria^23^, which has been adopted by the Chinese Society of Herpetology^24^. The degree of steatosis was assessed semi-quantitatively based on fatty-fibrotic patterns as described by Graif *et al.*^23^, and reported in our previous article^25^. In brief, the ultrasonic diagnosis was based on follows^25^: 1) diffuse enhancement of the near-field echo in the hepatic region (stronger than in the kidney and spleen regions) and gradual attenuation of the far-field echo; 2) unclear display of intra-hepatic lacuna structures; 3) mild to moderate hepatomegaly with a round and blunt border; 4) color Doppler ultrasonography showing a reduction of the blood flow signal in the liver or a difficult-to-display signal with a normal distribution of blood flow; and 5) unclear or non-intact display of the envelope of the right liver lobe and diaphragm. Subjects were graded into: absent (none of above items); mild (1 and any one of item 2–4); moderate (1 and any two of item 2–4); and severe (1, 5 and any two of item 2–4). NAFLD was then confirmed after excluding the following conditions: significant alcohol consumption for hepatic steatosis and chronic liver disease^1^.

### Statistical analysis

All analyses were conducted using SPSS statistical software (version 13.0, SPSS Inc., Chicago, IL), and P values of below 0.05 (two-tailed) were considered significant in all the statistical analyses. Skewed variables were natural logarithm (ln)-transformed before subsequent analyses. Analyses of variance (ANOVA) and covariance (ANCOVA) and t-tests were used to compare mean differences in the participants’ characteristics, and chi-square (or Fisher’s exact) tests were used to analyze differences in the frequencies of categorical variables among the NAFLD groups. ANOVA and ANCOVA were used to compare mean differences, and the sub-command of “*Polynomial contrasts*” was used to test the linear trend across the serum levels of TMAO, choline and betaine across the NAFLD groups in the CCS and CSS. Bonferroni *t* test was used for the multiple comparisons between the NAFLD groups. For the multivariate models, we adjusted for age, sex (in total men and women), waist circumference, SBP, blood cholesterol, triglyceride, HDLc, LDLc, glucose, uric acid, education levels (secondary or below, high school, college or above), job (light, moderate and heavy, in physcial labor work), household income (<4000, 4000-6000, >6000, yuan/month/person), smoking (current or /no), alcohol intake statuses (current or /no), physical activity (in MET h/week, excluding sleeping and sitting), and dietary intakes of total energy, fat, and fiber (continous variables except those defined). Logistic regression analyses were used to calculate the odds ratios (OR) and their 95% confidence interval (CI) of NAFLD (or related indices) for the quartiles of serum values of TMAO, betaine, and choline in the CSS, and for each one unit increase in ln-transformed serum values of TMAO, betaine and choline in the CCS.

## Results

### Case-control study

The NAFLD patients included 48 men and 12 women, with a mean (SD) age of 34.8 (10.2) years. There were 22 men and 13 women in the control group, with a mean age of 44.8 (10.8) years. The NAFLD patients had a younger mean age and higher levels of BMI, WC, DBP, and blood lipids (TC, TGs, LDLc, and HDLc) compared with the controls (all p < 0.05) (Table 1). The ln-transformed levels of TMAO were positively associated with the corresponding values of choline (*pearson correlation coefficient* (*r*) = 0.487, 0.203) and betaine (*r* = 0.294, 0.159) in the CCS and CSS (all p < 0.05) (data not shown).

ANOVA showed significantly higher scores for steatosis, NAS, and lobular inflammation in association with greater ln-transformed levels of TMAO, choline and betaine, except for the steatosis scores associated with betaine. The ratio of betaine to choline was inversely associated with the steatosis and NAS (all p < 0.05) (STable 1). After adjusting for the potential covariates (age, sex, smoking status, alcohol intake status, physical activity and WC), the associations were attenuated. However, significant associations remained between the serum ln-transformed level of TMAO, betaine/choline ratio, choline level, and steatosis and NAS (ANCOVA). The serum levels of betaine and choline were positively correlated with the lobular inflammation score. To determine whether the associations between the choline level and the scores for steatosis, NAS and lobular inflammation were mediated by TMAO, we further adjusted for TMAO in analysis of choline. The associations of choline with the steatosis, NAS and inflammation scores were attenuated to non- or marginally significant levels. (Table 2).

ORs (95%CI) of high score (≥1 *vs.* 0) of NAFLD indices for each one unit inrease in ln-transformed values of TMAO (μM) were 3.58 (1.38–9.26) for the statosis score, 3.34 (1.44–7.78) for the NASH score, and 2.37 (1.10–5.09) for the lobular inflammation score, respectively. Higher choline values were also associatied with greater risks of having high scores of statosis, NASH, and lobular inflmmation. (STable 3).

In consistent with the results NAFLD, ln-TMAO and ln-choline were positively and significantly correlated to serum levels of TC, TG and HDLc (all p < 0.05), but not to LDLc after adjusted for age and sex. (data not shown).

### Cross-sectional study

Table 3 shows the characteristics of the participants. Among 1628 participants, 43.2%, 10.3% and 2.6% had mild, moderate and severe NAFLD, respectively, and 79.4% were women. The severity of NAFLD was positively associated with age, BMI, WC, blood pressure, TGs, LDLc and glucose, but negatively correlated with HDLc in both the males and females (all p < 0.05). More NAFLD patients than normal subjects were smokers.

Table 4 shows that a higher concentration of ln-transformed TMAO and a lower concentration of ln-transformed betaine and betaine to choline ratio were associated with a greater severity of NAFLD after adjusting for age, sex, WC, metabolic risk factors, socioeconomic factors, smoking status and physical activity for all participants and for the women (all p-trend < 0.05). The associations were similar in women and in men. Univariate analysis resulted in more pronounced associations (STable 2). We did not observe any significant differences in ln-transformed the plasma choline concentration among NAFLD groups by either univariate or multivariate analysis of variance (Table 4, STable 2).

ORs (95%CI) of NAFLD (none *vs.* moderate or severe) for the highest (*vs.* lowest) quartile of TMAO, betaine and choline were 3.25 (1.61-6.56) , 0.13 (0.06-0.26) , and 0.47 (0.25–0.89), respectively. Similar trends were observed for the different cutoffs of NAFLD. (STable 4).

Positive association of ln-TAMO were observed with serum levels of TC, TG and LDLc (p < 0.05); while ln-betaine were inversely associated with TC, TG and LDLc (p < 0.05). No significant associations between ln-choline and lipids were observed . (data not shown).

## Discussion

### Main findings

In the present study, we found consistently adverse associations between the plasma TMAO level and the presence and severity of NAFLD in both the CCS and CSS and a favorable betaine-NAFLD association in the CSS. To our knowledge, the present study is the first to report the adverse association of TMAO with NAFLD in humans. Our findings suggest that TMAO, an intestinal microbiota-dependent metabolite of phosphatidylcholine (PC)/choline, may be an independent risk marker (possible risk factor) for NAFLD.

### TMAO and NAFLD

In recent years, a few studies have examined the association between TMAO and cardiovascular risk in both animals and humans. Wang *et al.*^14^ have identified a novel pathway (dietary PC/choline → gut-flora-formed TMA → hepatic-flavin monooxygenase-formed TMAO) linking the dietary intake of PC/choline, intestinal microflora and atherosclerosis in mice, in which TMAO promotes the upregulation of multiple macrophage scavenger receptors involved in atherosclerosis. This association has been further confirmed in humans^13^,^18^. Tang *et al.*^18^ have found that an increased TMAO level is correlated to an increased risk of incident MACE (HR for quartile 4 vs. 1: 2.54, 95%CI: 1.96-3.28) in a 3-year follow-up study of 4007 patients who underwent elective coronary angiography. In addition, an elevated plasma TMAO level has been associated with a 2.2-fold (95%CI, 1.42–3.43) increased risk of mortality after adjusting for traditional risk factors in 720 patients with stable heart failure^26^. Similar results have been observed in other studies^27^,^28^. Further, TMAO may decrease long-term survival in stable subjects with chronic kidney disease, and contribute to progressive renal fibrosis and dysfunction in a murine model^29^. However, limited data are available for NAFLD. Dumas *et al.*^30^ have found that a high urinary excretion of TMAO is associated with insulin resistance and NAFLD in mice (129S6) prone to these diseases. In agreement with these previous studies, we showed an adverse association of the plasma TMAO level with the presence and severity of NAFLD in both clinical patients and community-based participants. The findings of our study and previous studies suggested that TMAO might play diverse adverse roles in the development and progression of various chronic diseases.

The increased risk of fatty liver disease might be caused by TMAO due to its effect on decreasing the total bile acid pool size^16^ via the following mechanisms: 1) by decreasing the synthesis of bile acids due to the inhibition of the key enzymes CYP7A1 and CYP27A1^16^; and 2) by limiting the enterohepatic circulation of bile acids between the liver and intestines due to the repression of organic anion transporter (oapt) and multidrug resistance protein (MRP) family protein expression^16^. In addition to their well-established roles in dietary lipid absorption and cholesterol homeostasis, bile acids also act as metabolically active signaling molecules to modulate glucose and lipid metabolism. Therefore, it is possible that TMAO may affect hepatic TGs levels, and reverse the directions of cholesterol transport and glucose and energy homeostasis by altering the synthesis and transport of bile acids, indicating that it is potential risk factor for fatty liver disease.

### Choline and NAFLD

Choline is a constituent of cell and mitochondrial membranes. Choline deficiency may affect liver from steatosis to carcinomas via diverse processes, including abnormal phospholipid synthesis, defective very-low-density lipoprotein (VLDL) secretion, aberrations in the methylation-dependent biosynthesis of molecules and the enterohepatic circulation of bile and cholesterol, etc.^8^. A large number of studies have demonstrated that a choline-deficient diet may cause NAFLD in animals and humans^8^,^31^,^32^. It has been reported that a low dietary choline intake is associated both with a higher risk of NAFLD in normal-weight Chinese women^33^ and with worse liver fibrosis in postmenopausal US women with NAFLD^9^. On the other hand, high plasma choline levels have been associated with an unfavorable cardiovascular risk factor profile^11^ and higher MACE risk^12^,^13^. To our knowledge, only one study has examined circulating choline levels and NAFLD, showing that plasma free choline levels are positively related to the severity of liver steatosis, fibrosis and NASH in Japanese^10^. In this study, we observed an unfavorable association in the hospital-based patients, but not in the community-based adults. The adverse association between circulating choline and NAFLD detected in the CCS may have been due to the TMAO level because this significant association was almost attenuated to null after analysis was adjusted for TMAO.

### Betaine and NAFLD

Betaine is an important methyl donor that may be obtained from dietary sources or synthesized endogenously from choline. It may supply its methyl group for the remethylation of homocysteine (Hcy) to methionine and the further regeneration of S-adenosylmethionine (SAM). SAM may subsequently facilitate the metabolic conversion of phosphatidylethanolamine (PE) to form Phosphatidylcholine (PC). Plenty of betaine may partially spare choline^34^ which is a basic component for the synthesis of PC. PC is necessary for the packaging of VLDL and then promoting lipid exportation from the liver^35^, subsequently attenuating fatty liver^36^. Several animal studies have shown that betaine supplementation protects the liver from fat accumulation in rodent models induced by high-fat diets^37^,^38^. A small number of randomized controlled trials (RCT) have also reported such beneficial effects of betaine. In a short-term RCT, betaine supplementation for 8 weeks has been found to reduce hepatic steatosis by 25% and attenuate the hepatic concentrations of AST and ALT in 191 patients with NASH^39^. Another RCT has shown that betaine treatment (20 g/d vs. placebo) for 12 months improves hepatic steatosis and may protect against the worsening of steatosis in 55 patients with biopsy-proven NASH^40^. Consistent with these results, we observed a significantly inverse association between the plasma betaine concentration and the severity of NAFLD in 1628 community-based adults. Due to the limited evidence in humans, further prospective studies are needed to confirm the potential benefits of betaine in NAFLD.

### Strengths and limitations

The strengths of our study include the following: we examined the associations of the plasma levels of TMAO, choline and betaine with NAFLD in both clinical patients with/without biopsy-proven NAFLD and in community-based adults evaluated by ultrasound. In addition, this is the first report of a consistently adverse association between TMAO and NAFLD. Liver biopsy, which was performed in the CCS, is the gold standard for evaluating hepatic steatosis. In addition, the relatively large study size allowed us to have higher power to determine these associations in the CSS, and a variety of covariates including various cardio-metabolic risk factors were adjusted for to avoid potential confounding biases in both studies. However, some residual confounders might be existed due to unmeasured covariates or the potential measurement errors in our studies.

Several limitations should be acknowledged. First, we could not infer a causal relationship between TMAO and NAFLD due to the limitations associated with the study designs used. In addition, we measured the plasma levels of choline and its metabolites in samples obtained at baseline and assessed NAFLD at the follow-up in the CSS. The reported associations are dependent on the stability of the blood levels of TMAO, choline and betaine over an interval of 3.2 years. However, the potential changes in the relevant blood concentrations over the 3.2 years tended to dilute (and not increase) the reported associations, and the delayed evaluation of the dependent variable decreased the possibility of inverse causality in the CSS. Third, liver tissue evaluation is the gold standard method for the assessment of fatty live. Ultrasonography conducted in our CSS may be less sensitive than magnetic resonance spectroscopy in comparison to histology^41^. A meta-analysis in 2011 reported that liver ultrasound has acceptable sensitivity (84%, 95%CI: 0.79%−0.89%) and excellent specificity (94%, 95%CI: 0.87%−0.97%) compared with liver biopsy^42^. The misclassification caused by relatively low sensitivity of ultrasound at a low liver fat content^41^ was unlikely to overestimate the observed association in this study. Fourth, a limited study size was used for the biopsy-proven NAFLD cases and controls due to the difficulty in obtaining such samples. Fifth, inconsistent results for the betaine and choline levels were observed in the CCS and CSS, likely due to the different populations assessed. Lastly, we could not determine whether TMAO was a risk factor or just a marker of co-existing risk factor(s). Howevr, our findings provided a useful clue for further studies of identification of NAFLD causes in humans.

## Conclusions

Our findings showed an inverse association of circulating TMAO level with the presence and severity of NAFLD in both the clinical patients and community-based adults, and a favorable relationship between the blood betaine concentration and the severity of NAFLD in the community-based participants but not in the clinical patients. Further studies, particularly inventional human studies or animal models, are needed to clarify if there is a causal relationship between TMAO and NAFLD.

## Additional Information

**How to cite this article**: Chen, Y.-m. *et al.* Associations of gut-flora-dependent metabolite trimethylamine-N-oxide, betaine and choline with non-alcoholic fatty liver disease in adults. *Sci. Rep.* **6**, 19076; doi: 10.1038/srep19076 (2016).

## Supplementary Material

Supplementary Information

## Figures and Tables

**Figure 1:**
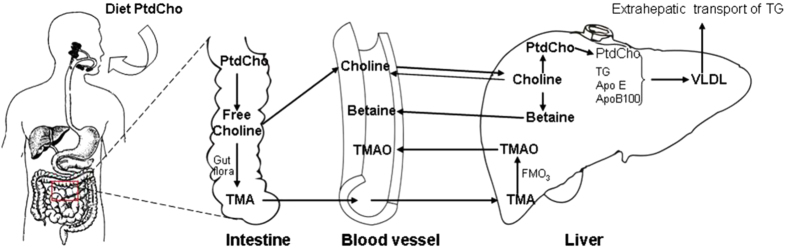
Schematic chart of choline metabolism. The chart was drawn by Y.L. and H.L.Z. Abbreviations: PtdCho: phosphatidylcholine; TG: Triglyceride; TMA: Trimethylamine; TMAO: Trimethylamine N-oxide; VLDL: Very low density lipoprotein.

**Table 1:** Characteristics of the study population in the case-control study^1^.

	Normal subjects (n = 35)	NAFLD patients (n = 60)	*P*	Adjusted *P*^1^	Adjusted *P*^*2*^
General characteristics					
Age, years	44.8 ± 10.8	34.8 ± 10.2	**<0.001**	–	–
Male/female, n	22/13	48/12	0.067	–	–
BMI, kg/m^2^	22.6 ± 3.2	27.9 ± 3.1	**<0.001**	**<0.001**	–
Waist circumference, cm	84.6 ± 9.1	95.6 ± 7.4	**<0.001**	**<0.001**	–
SBP, mmHg	120.8 ± 11.4	125.4 ± 13.5	0.117	0.180	0.892
DBP, mmHg	78.0 ± 9.3	82.6 ± 10.9	**0.044**	0.131	0.472
Current smoker, n(%)	10(30.3)	17(29.8)	0.962	0.470	–
Passive smoker, n(%)	17(56.67)	30(56.60)	0.996	0.393	–
Physical activity, MET	37.7 ± 21.1	35.3 ± 23.4	0.619	0.269	–
Biochemical characteristics					
AST, U/L	26.0(20.7,44.5)	30.1(22.6,42.1)	0.871	**0.049**	0.951
ALT, U/L	41.8(24.0,73.6)	56.5(31.1,87.7)	0.206	0.321	0.807
Blood glucose, mg/dL	5.30(4.97,5.85)	5.09(4.76,5.95)	0.502	0.215	0.344
Triglycerides, mmol/L	1.15(0.76,1.69)	1.79(1.18,2.71)	**0.005**	**0.039**	0.925
Cholesterol, mmol/L	3.95 ± 1.50	5.22 ± 1.13	**<0.001**	**<0.001**	0.062
HDLc, mg/dL	1.00 ± 0.31	1.25 ± 0.26	**<0.001**	**0.001**	0.140
LDLc, mg/dL	2.49 ± 1.02	2.92 ± 0.83	**0.028**	**0.025**	0.152
HBV( + ), n(%)	12(35.3)	16(27.1)	0.659	0.122	–

^1^The data are expressed as the mean ± SD or median (interquartile range) according to the distribution of variables.

The *P* values in bold indicates significant differences. Adjusted *P*^1^: Adjusted for age and gender by analysis of covariance (ANCOVA) for continous variables, and multivariate logistic regression analysis for binary variables. Adjusted *P*^2^: Adjusted for age, gender, HB virus, MET, BMI, waist circumference, and smoking status by using ANCOVA or logistic regression analysis. Abbreviations: ALT: alanine aminotransferase; AST: aspartate aminotransferase; BMI: body mass index; DBP: diastolic blood pressure; SBP: systolic blood pressure; HDLc: high-density lipoprotein cholesterol; LDLc: low-density lipoprotein cholesterol; and MET: metabolic equivalent task.

**Table 2:** Comparison of the covariate-adjusted means of serum TMAO, betaine, choline and betaine to choline ratio according to histologic features of NAFLD in the case-control study.

	n	mean	SE	n	mean	SE	n	mean	SE	ANCOVA	
										*P*_-Diff_	*P*_-trend_
**Steatosis score**		**0**			**1**			**2–3**			
Ln(TMAO, μM)	35	2.20	0.20	31	3.09	0.18**	25	2.95	0.22	**0.011**	0.026
Ln(Betaine, μM)	35	3.553	0.77	31	3.552	0.071	25	3.566	0.085	0.990	0.920
Ln(Choline, μM)	35	2.209	0.087	31	2.444	0.080	25	2.543	0.096	0.072	**0.025**
Ln(Choline, μM) #		2.308	0.082		2.371	0.075		2.496	0.088	0.346	0.174
Betaine/Choline	35	1.344	0.083	31	1.107	0.076	25	1.023	0.091	0.063	**0.023**
Betaine/Choline#		1.313	0.086		1.131	0.078		1.038	0.091	0.160	0.058
**NAFLD activity score**		**0**			**1–2**			**3–5**			
Ln(TMAO, μM)	23	1.767	0.245	28	2.963	**	40	3.068	0.157**	**<0.001**	**<0.001**
Ln(Betaine, μM)	23	3.384	0.098	28	3.625	0.071	40	3.608	0.063	0.160	0.089
Ln(Choline, μM)	23	1.929	0.103	28	2.488	0.075**	40	2.566	0.066**	**<0.001**	**<0.001**
Ln(Choline, μM) #		2.166	0.109		2.474	0.081		2.435	0.066	0.104	0.065
Betaine/Choline	23	1.455	0.106	28	1.137	0.077	40	1.041	0.068*	**0.016**	**0.004**
Betaine/Choline#		1.419	0.116		1.147	0.078		1.055	0.071	0.071	**0.022**
**Lobular inflammation score**		**0**			**1**			**2–3**			
Ln(TMAO, μM)	30	2.196	0.216	33	3.169	0.193*	26	2.667	0.194	**0.015**	0.128
Ln(Betaine, μM)	30	3.338	0.079	33	3.662	0.070*	26	3.640	0.071*	**0.015**	**0.009**
Ln(Choline, μM)	30	2.058	0.090	33	2.600	0.080^**^	26	2.469	0.081^**^	**0.001**	**0.002**
Ln(Choline, μM) #		2.180	0.088		2.507	0.082>*		2.428	0.075	**0.054**	**0.042**
Betaine/Choline	30	1.280	0.092	33	1.062	0.082	26	1.172	0.083	0.303	0.407
Betaine/Choline#		1.239	0.094		1.101	0.085		1.169	0.082	0.647	0.595

^*,**^compared with histologic features of “0” group; *P* < 0.05 and *P* < 0.01, respectively.

ANCOVA (analysis of covariance): adjusted for age, sex, smoking status (yes/no), alcohol intake status (yes/no), physical activity (in MET [hour/day], excluding sleeping and sitting), and waist circumference. Bonferroni *t* test was used for the multiple comparisons between the NAFLD groups. P values of below 0.05 (two-tailed) were considered significant.

*P-Diff*: P value for the difference among the groups.

#: further adjusted for ln-TMAO.

**Table 3:**
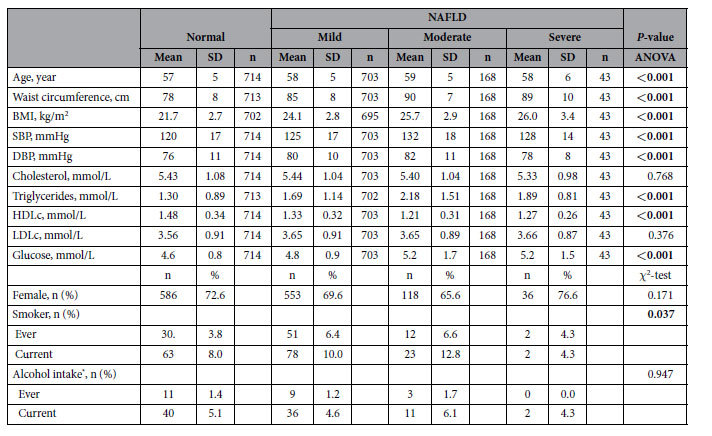
Characteristics of the study participants in the cross-sectional study (CSS)^1^.

^1^The data are expressed as the mean ± SD or n (%) according to the measurements or frequencies of the variables.

The *P* values in bold indicate significant differences. Abbreviations: BMI: body mass index; DBP: diastolic blood pressure; SBP: systolic blood pressure; HDLc: high-density lipoprotein cholesterol; and LDLc: low-density lipoprotein cholesterol. ^*^Drinking alcohol beverages ≥1 times/week in the past year. The fisher’s exact test was used.

**Table 4:** Comparison of covariate-adjusted means of serum TMAO, betaine, choline and betaine to choline ratio with NAFLD groups in the cross-sectional study.

	NAFLD										
	Normal			Mild			Moderate & Severe			ANCOVA	
	n	Mean	SE	n	Mean	SE	n	Mean	SE	*P*_-Diff_	*P*_-trend_
Total											
Ln(TMAO, μM)	643	0.104	0.040	643	0.160	0.038	197	0.434	0.073**,##	**<0.001**	**<0.001**
Ln(Betaine, μM)	662	3.840	0.017	671	3.809	0.016	208	3.667	0.030**,##	**<0.001**	**<0.001**
Ln(Choline, μM)	662	3.179	0.018	671	3.169	0.017	208	3.092	0.032	0.065	**0.026**
Betaine/Choline	662	2.156	0.037	671	2.096	0.035	208	1.987	0.065	0.104	**0.034**
Women											
Ln(TMAO, μM)	457	0.096	0.049	437	0.134	0.047	135	0.382	0.089*	**0.021**	**0.007**
Ln(Betaine, μM)	470	3.792	0.021	453	3.755	0.020	140	3.601	0.038**,##	**<0.001**	**<0.001**
Ln(Choline, μM)	470	3.165	0.022	453	3.158	0.022	140	3.071	0.041	0.120	0.053
Betaine/Choline	470	2.084	0.042	453	2.010	0.041	140	1.930	0.077	0.243	0.106
Men											
Ln(TMAO, μM)	186	0.118	0.075	206	0.222	0.066	62	0.539	0.130*	0.032	**0.009**
Ln(Betaine, μM)	192	3.948	0.028	218	3.925	0.025	68	3.822	0.048	0.096	0.035
Ln(Choline, μM)	192	3.208	0.030	218	3.188	0.027	68	3.154	0.051	0.699	0.399
Betaine/Choline	192	2.325	0.074	218	2.287	0.064	68	2.116	0.124	0.377	0.175

^*,**^compared with “Normal”; *P* < 0.05 and *P* < 0.01, respectively;

^##^compared with “Mild NAFLD”; *P* < 0.01, respectively.

The following covariates were adjusted for: age, sex (male/female) (in total), waist circumference, SBP, blood cholesterol, triglyceride, HDL, LDL glucose, uric acid, and education levels (secondary or below, high school, college or above), job (light, moderate and heavy, in physcial labor work), household income (<4000, 4000-6000, >6000, yuan/month/person), smoking (yes/no), and alcohol intake statuses (yes /no), and physical activity (in MET h/week, excluding sleeping and sitting), and dietary intakes of total energy, fat, and fiber (continous variables except those defined). Bonferroni *t* test was used for the multiple comparisons between the NAFLD groups. P values of below 0.05 (two-tailed) were considered significant.
