# Supplementary material for: Associations of gut-flora-dependent metabolite trimethylamine-N-oxide, betaine and choline with non-alcoholic fatty liver disease in adults
Source: Sci Rep. 2016 Jan 8;6:19076. doi: 10.1038/srep19076 (PMC4705470; doi:10.1038/srep19076)
Supplement: Supplementary Information [file srep19076-s1.doc]

**Associations of gut-flora-dependent metabolite trimethylamine-N-oxide, betaine and choline with non-alcoholic fatty liver disease in adults**

Yu-ming Chen ^1,#^, Yan Liu ^1,#^, Rui-fen Zhou ^1^, Xiao-ling Chen ^1^, Cheng Wang ^1^, Xu-ying Tan^1^, Li-jun Wang ^2^, Rui-dan Zheng ^3^, Hong-wei Zhang ^4^, Wen-hua Ling ^1^, Hui-lian Zhu ^1,*^

**Supplemental Tables**

**STable 1**

Comparison of univariate means of serum TMAO, betaine, choline and betaine to choline ratio with the histologic features of NAFLD in the case-control study

|  | n | mean | SE |  | n | mean | SE |  | n | mean | SE |  | ANOVA  p-value |
| --- | --- | --- | --- | --- | --- | --- | --- | --- | --- | --- | --- | --- | --- |
| **Steatosis score** |  | **0** |  |  |  | **1** |  |  |  | **2-3** |  |  |  |
| Ln(TMAO, µM) | 35 | 2.188 | 0.187 |  | 31 | 3.146 | 0.139^**^ |  | 25 | 2.888 | 0.187^**^ |  | **<0.001** |
| Ln(Betaine, µM) | 35 | 3.495 | 0.078 |  | 31 | 3.590 | 0.047 |  | 25 | 3.601 | 0.048 |  | 0.411 |
| Ln(Choline, µM) | 35 | 2.116 | 0.080 |  | 31 | 2.508 | 0.044 |  | 25 | 2.596 | 0.095 |  | **<0.001** |
| Betaine/Choline | 35 | 1.379 | 0.057 |  | 31 | 1.082 | 0.060^**^ |  | 25 | 1.005 | 0.101^**^ |  | **0.001** |
|  |  |  |  |  |  |  |  |  |  |  |  |  |  |
| **Total NAFLD activity score** |  | **0** |  |  |  | **1-2** |  |  |  | **3-5** |  |  |  |
| Ln(TMAO, µM) | 23 | 1.853 | 0.213 |  | 28 | 2.975 | 0.173^**^ |  | 40 | 3.010 | 0.107**^**^** |  | **<0.001** |
| Ln(Betaine, µM) | 23 | 3.382 | 0.108 |  | 28 | 3.638 | 0.052^**^ |  | 40 | 3.599 | 0.035^*^ |  | **0.017** |
| Ln(Choline, µM) | 23 | 1.918 | 0.098 |  | 28 | 2.526 | 0.042^**^ |  | 40 | 2.546 | 0.064^**^ |  | **<0.001** |
| Betaine/Choline | 23 | 1.464 | 0.077 |  | 28 | 1.112 | 0.064^**^ |  | 40 | 1.053 | 0.067^**^ |  | **<0.001** |
|  |  |  |  |  |  |  |  |  |  |  |  |  |  |
| **Lobular inflammation score** |  | **0** |  |  |  | **1** |  |  |  | **2-3** |  |  |  |
| Ln(TMAO, µM) | 30 | 2.173 | 0.200 |  | 33 | 3.117 | 0.136^**^ |  | 26 | 2.761 | 0.159^*^ |  | **<0.001** |
| Ln(Betaine, µM) | 30 | 3.360 | 0.085 |  | 33 | 3.647 | 0.038^**^ |  | 26 | 3.634 | 0.041**^**^** |  | **0.001** |
| Ln(Choline, µM) | 30 | 2.061 | 0.093 |  | 33 | 2.577 | 0.074 |  | 26 | 2.495 | 0.041 |  | **<0.001** |
| Betaine/Choline | 30 | 1.300 | 0.085 |  | 33 | 1.071 | 0.081^*^ |  | 26 | 1.139 | 0.044 |  | 0.088 |
|  |  |  |  |  |  |  |  |  |  |  |  |  |  |
| **Fibrosis score** |  | **0** |  |  |  | **1-4** |  |  |  |  |  |  |  |
| Ln(TMAO, µM) | 48 | 2.469 | 0.152 |  | 43 | 2.972 | 0.124 |  |  |  |  |  | **0.012** |
| Ln(Betaine, µM) | 48 | 3.487 | 0.060 |  | 43 | 3.634 | 0.036 |  |  |  |  |  | **0.039** |
| Ln(Choline, µM) | 48 | 2.388 | 0.085 |  | 43 | 2.485 | 0.035 |  |  |  |  |  | **0.036** |
| Betaine/Choline | 48 | 1.199 | 0.074 |  | 43 | 1.149 | 0.043 |  |  |  |  |  | 0.575 |

*, **: compared with histologic features of “0” group; *P* <0.05 and *P* <0.01, respectively.

**STable 2**

Comparison of means of serum TMAO, betaine, choline and betaine to choline ratio according to NAFLD categories in the cross-sectional study

|  | Normal | | |  | NAFLD | | | | | | | | | | |  | ANOVA | |
| --- | --- | --- | --- | --- | --- | --- | --- | --- | --- | --- | --- | --- | --- | --- | --- | --- | --- | --- |
|  |  |  |  |  | Mild | | |  | Moderate | | |  | Server | | |  |  |  |
|  | n | Mean | SD |  | n | Mean | SD |  | n | Mean | SD |  | n | Mean | SD |  | *P*_-Diff_ | *P*_-trend_ |
| Total |  |  |  |  |  |  |  |  |  |  |  |  |  |  |  |  |  |  |
| Ln(TMAO, µM) | 673 | 0.124 | 1.005 |  | 666 | 0.152 | 0.936 |  | 158 | 0.308 | 0.857 |  | 42 | 0.719 | 0.965**^**,##^** |  | **<0.001** | **<0.001** |
| Ln(Betaine, µM) | 694 | 3.837 | 0.427 |  | 696 | 3.808 | 0.406 |  | 167 | 3.666 | 0.453**^**,##^** |  | 43 | 3.585 | 0.509**^**,##^** |  | **<0.001** | **<0.001** |
| Ln(Choline, µM) | 694 | 3.168 | 0.457 |  | 696 | 3.167 | 0.414 |  | 167 | 3.076 | 0.379 |  | 43 | 3.121 | 0.472 |  | 0.071 | 0.061 |
| Betaine/Choline | 694 | 2.184 | 0.922 |  | 696 | 2.092 | 0.869 |  | 167 | 1.990 | 0.831 |  | 43 | 1.824 | 0.889**^*^** |  | **0.006** | **<0.001** |
| Women |  |  |  |  |  |  |  |  |  |  |  |  |  |  |  |  |  |  |
| Ln(TMAO, µM) | 471 | 0.118 | 1.023 |  | 453 | 0.115 | 0.932 |  | 109 | 0.216 | 0.825 |  | 32 | 0.726 | 1.067**^**,##^** |  | **0.005** | **0.017** |
| Ln(Betaine, µM) | 487 | 3.782 | 0.445 |  | 471 | 3.756 | 0.409 |  | 109 | 3.617 | 0.458**^**,#^** |  | 32 | 3.513 | 0.535**^**#^** |  | **<0.001** | **<0.001** |
| Ln(Choline, µM) | 487 | 3.149 | 0.459 |  | 471 | 3.159 | 0.438 |  | 109 | 3.052 | 0.411 |  | 32 | 3.153 | 0.526 |  | 0.156 | 0.276 |
| Betaine/Choline | 487 | 2.112 | 0.905 |  | 471 | 2.008 | 0.812 |  | 109 | 1.953 | 0.812 |  | 32 | 1.680 | 0.935**^*^** |  | **0.013** | **0.002** |
| Men |  |  |  |  |  |  |  |  |  |  |  |  |  |  |  |  |  |  |
| Ln(TMAO, µM) | 202 | 0.138 | 0.964 |  | 213 | 0.232 | 0.939 |  | 53 | 0.490 | 0.915 |  | 10 | 0.697 | 0.731 |  | **0.040** | **0.006** |
| Ln(Betaine, µM) | 207 | 3.966 | 0.349 |  | 225 | 3.914 | 0.379 |  | 58 | 3.759 | 0.432**^**,#^** |  | 11 | 3.796 | 0.370 |  | **0.002** | **<0.001** |
| Ln(Choline, µM) | 207 | 3.211 | 0.450 |  | 225 | 3.184 | 0.359 |  | 58 | 3.122 | 0.307 |  | 11 | 3.028 | 0.253 |  | 0.241 | 0.054 |
| Betaine/Choline | 207 | 2.352 | 0.941 |  | 225 | 2.269 | 0.956 |  | 58 | 2.058 | 0.868 |  | 11 | 2.241 | 0.588 |  | 0.207 | 0.063 |

*, **: compared with “Normal”; *P* <0.05 and *P*<0.01, respectively;

#, ##, compared with “Mild NAFLD”; *P*<0.05 and *P*<0.01, respectively.

|  | OR1 | 95%CI | | p |  | OR2 | 95%CI | | p |
| --- | --- | --- | --- | --- | --- | --- | --- | --- | --- |
| **Steatosis score** | **0-1 *vs.* 2-3** | | | |  | **0 *vs.* 1-3** | | | |
| Ln(TMAO, µM) | 1.22 | 0.69 | 2.14 | 0.489 |  | 3.58 | 1.38 | 9.26 | 0.009 |
| Ln(Betaine, µM) | 1.09 | 0.21 | 5.62 | 0.921 |  | 0.42 | 0.07 | 2.51 | 0.343 |
| Ln(Choline, µM) | 5.21 | 1.02 | 26.60 | 0.047 |  | 7.09 | 0.95 | 52.8 | 0.056 |
| **NASH score** | **0-2 *vs.* 3-5** | | | |  | **0 *vs.* 1-5** | | | |
| Ln(TMAO, µM) | 1.82 | 1.02 | 3.24 | 0.042 |  | 3.34 | 1.44 | 7.78 | 0.007 |
| Ln(Betaine, µM) | 1.22 | 0.31 | 4.90 | 0.778 |  | 5.19 | 0.74 | 36.45 | 0.098 |
| Ln(Choline, µM) | 4.18 | 1.03 | 17.02 | 0.046 |  | 62.00 | 4.79 | 802.56 | 0.002 |
| **Lobular inflammation score** | **0-1 *vs.* 2-3** | | | |  | **0 *vs.* 1-3** | | | |
| Ln(TMAO, µM) | 0.96 | 0.57 | 1.61 | 0.870 |  | 2.37 | 1.10 | 5.09 | 0.027 |
| Ln(Betaine, µM) | 3.34 | 0.65 | 17.21 | 0.150 |  | 11.49 | 1.51 | 87.45 | 0.018 |
| Ln(Choline, µM) | 1.71 | 0.56 | 5.18 | 0.344 |  | 24.61 | 2.89 | 209.53 | 0.003 |

**STable 3.** Multivariate-adjusted Odds ratios of NAFLD scores for serum levels of TMAO, betaine and choline in the case-control study

Covariates adjusted for: age, sex, smoking status, physical activity, and waist circumference.

**STable 4**. Multivariate-adjusted Odds ratios of NAFLD for quartiles of serum levels of TMAO, betaine and choline in the cross-sectional study

|  | Q1 |  | Q2 | | |  | Q3 | | |  | Q4(highest) | | |  | p-diff. | p-trend |
| --- | --- | --- | --- | --- | --- | --- | --- | --- | --- | --- | --- | --- | --- | --- | --- | --- |
|  | OR |  | OR | 95%CI | |  | OR | 95%CI | |  | OR | 95%CI | |  |  |  |
| **NAFLD1** (0= none, 1=mild, moderate and severe NAFLD) | | | | | | | | | | | | | | | | |
| TMAO | 1.00 |  | 1.18 | 0.84 | 1.66 |  | 1.18 | 0.85 | 1.64 |  | 1.53 | 1.08 | 2.17 |  | 0.113 | 0.023 |
| Betaine | 1.00 |  | 0.78 | 0.56 | 1.10 |  | 0.74 | 0.53 | 1.03 |  | 0.62 | 0.44 | 0.87 |  | 0.045 | 0.006 |
| Choline | 1.00 |  | 1.05 | 0.76 | 1.46 |  | 1.08 | 0.77 | 1.50 |  | 1.05 | 0.76 | 1.47 |  | 0.976 | 0.735 |
| B/C | 1.00 |  | 0.97 | 0.69 | 1.35 |  | 0.79 | 0.57 | 1.09 |  | 0.72 | 0.51 | 1.00 |  | 0.148 | 0.025 |
| **NAFLD2** (0= none & mild NAFLD, 1= moderate & severe NAFLD) | | | | | | | | | | | | | | | | |
| TMAO | 1.00 |  | 2.00 | 1.16 | 3.42 |  | 2.24 | 1.34 | 3.74 |  | 2.62 | 1.53 | 4.50 |  | 0.004 | <0.001 |
| Betaine | 1.00 |  | 0.68 | 0.45 | 1.02 |  | 0.34 | 0.21 | 0.56 |  | 0.25 | 0.14 | 0.43 |  | <0.001 | <0.001 |
| Choline | 1.00 |  | 0.74 | 0.47 | 1.16 |  | 0.88 | 0.57 | 1.36 |  | 0.46 | 0.28 | 0.75 |  | 0.016 | 0.009 |
| B/C | 1.00 |  | 0.89 | 0.58 | 1.37 |  | 0.65 | 0.41 | 1.03 |  | 0.71 | 0.44 | 1.15 |  | 0.241 | 0.072 |
| **NAFLD3** (0= none, 1= moderate and severe NAFLD) | | | | | | | | | | | | | | | | |
| TMAO | 1.00 |  | 2.19 | 1.10 | 4.36 |  | 2.41 | 1.24 | 4.69 |  | 3.25 | 1.61 | 6.56 |  | 0.010 | <0.001 |
| Betaine | 1.00 |  | 0.56 | 0.32 | 0.96 |  | 0.27 | 0.14 | 0.52 |  | 0.13 | 0.06 | 0.26 |  | <0.001 | <0.001 |
| Choline | 1.00 |  | 0.91 | 0.51 | 1.62 |  | 1.11 | 0.63 | 1.96 |  | 0.53 | 0.28 | 1.01 |  | 0.134 | 0.145 |
| B/C | 1.00 |  | 1.01 | 0.57 | 1.80 |  | 0.61 | 0.33 | 1.11 |  | 0.47 | 0.25 | 0.89 |  | 0.037 | 0.006 |

B/C: betaine to choline ratio.

OR, 95%CI: Odds ratio and 95% confidence interval calculated using multivariate logistic regress. Covariates adjuste for: age, sex (in total), waist circumference, SBP, blood cholesterol, triglyceride, HDL, LDL glucose, uric acid, and education levels, job, economic, smoking, and alcohol intake statuses, and physical activity (in MET, excluding sleeping and sitting), and dietary intakes of total energy, fat, and fiber.

p-diff., p-value for the quartile differences; p-trend, p-value for linear trend, calculated by treating the quartile numbers as continous variable.
